# Temporal Changes in Functional Magnetic Resonance Imaging Activation of Heterosexual Couples for Visual Stimuli of Loved Partners

**DOI:** 10.4306/pi.2009.6.1.19

**Published:** 2009-03-31

**Authors:** Won Kim, Seungyeon Kim, Jaeseung Jeong, Kyung-Uk Lee, Kook-Jin Ahn, Yong-An Chung, Keun-Young Hong, Jeong-Ho Chae

**Affiliations:** 1 Department of Psychiatry and Stress Research Institute, Seoul Paik Hospital, College of Medicine, Inje University, Seoul, Korea.; 2 Department of Bio and Brain Engineering, Korea Institute of Science and Technology (KAIST), Daejeon, Korea.; 3 Department of Psychiatry, College of Medicine, The Catholic University of Korea, Seoul, Korea.; 4 Department of Radiology and Nuclear Medicine, College of Medicine, The Catholic University of Korea, Seoul, Korea.

**Keywords:** Functional magnetic resonance imaging, Blood oxygen level dependent, Romantic love, Temporal changes

## Abstract

**Objective:**

Previous neuroimaging studies on romantic love have focused on determining how the visual stimuli that serve as a representation of loved ones induce the neural activation patterns of romantic love. The purpose of this study was to investigate the temporal changes in romantic love over a period of 6 months and their correlated neurophysiological changes.

**Methods:**

Five heterosexual couples (n=10, mean age 21.1±1.97) who started dating not less than 100 days previously were recruited to measure their blood oxygen level dependent (BOLD) signals using functional magnetic resonance imaging (fMRI) while showing them pictures of their loved ones and their previously identified, opposite-sex friends. Subsequently, the subjects were scanned under the same experimental conditions to assess possible changes in their brain activities after 180 days.

**Results:**

We found that their Passionate Love Score (PLS) values (M: 118.6±9.1, F: 120.2±7.0) were significantly reduced after 6 months (M: 110.8±4.0, F: 106.2±3.0). Furthermore, significantly increased activations were found in the cingulate gyri, inferior frontal gyri, supramarginal gyri, etc., after 6 months, whereas the head and tail of the right caudate nucleus were deactivated, which is indicative of the inhibition of expression and sensory neglect.

**Conclusion:**

These findings suggest that dynamic neural processes in the cortical-subcortical regions are involved in temporal changes in romantic love.

## Introduction

Romantic love is often simply associated with human sexual activity for mate choices and the willing, and even eager, participation of the individuals, and yet it is a profound emotion that prevails in human activity throughout the world.^1^ Romantic love is frequently described as a highly motivating and rewarding experience.^2^,^3^ Persons who experience romantic love are typically highly energetic with their attention focused on their lovers, although they are often subject to mood swings as well.^3^,^4^ The physiological characteristics of romantic love include sweaty palms and a pounding heart, which indicate the existence of anxiety when their lover is not present. Persons who fall in love frequently exhibit an emotional dependence in which they rely on their loved ones for emotional support and they at times will go so far as to change themselves to impress their beloved.^3^

The first work on the neural basis of romantic love suggested that distinct networks of brain areas are responsible, such as the anterior cingulate gyrus and caudate nucleus.^5^ Subsequently, Bartels and Zeki^5^,^6^ investigated the neural correlates of both maternal and romantic love and found that they commonly activate the mesolimbic reward system that coincides with areas rich in oxytocin and vasopressin receptors. Their studies demonstrated that the critical social assessments and negative emotions of the persons were deactivated while they were in romantic love. On the other hand, Aron et al.^2^ reported using functional magnetic resonance imaging (fMRI) and other physiological measurements that, in an early stage, intense romantic love is associated with subcortical reward regions rich in dopamine, and that the early stages of romantic love also engage brain systems that are associated with the motivation to acquire further reward. They suggested that romantic love does not have any evident, specialized circuitry for its functions, but rather, activates collections of neural systems converging in the caudate region, where the specialized integration of many stimuli has previously been reported.^2^,^7^,^8^

Regions known to have rich vasopressin and oxytocin receptor levels in the human brain are activated by both maternal and romantic love.^6^,^9^ Oxytocin is associated with the functions of the parasympathetic component of the autonomic nervous system. It also plays a major role in the muscular contractions necessary for birth and lactation.^10^ Recently, the roles of oxytocin have been extended to include human trust^11^ and the modulation of social cognition and fear.^14^ The functions of vasopressin are reported to be peripheral activity attention and varying learning and memory functions.^12^,^13^ Both vasopressin and oxytocin affect social behavior.^14^,^15^

Although romantic love is a dynamic process, neuroimaging studies on temporal changes in neural activity during romantic love have not yet been performed. The aim of this work was to investigate the neural substrates at an early stage of romantic love within 100 days of its inception using fMRI and to determine any temporal changes in neural activities after 180 days compared with the early stages of love. This study was initiated at the request of the Korea Broadcasting System (KBS), Korea's public broadcaster, for a nationally televised documentary film entitled "Love". Part of this study, particularly the experimental procedure and the result, appeared in this film and was broadcast nationally in 2006.

## Methods

### Subjects

Five heterosexual, right-handed couples exhibiting passionate love for each other (n=10, mean age: 21.1±1.97 years, range: 18-24 years) were recruited by advertisements placed by KBS. We screened approximately 200 volunteers after initial rounds of professional interviews by psychologists. They were deemed to be psychologically, medically and neurologically healthy, as determined by the diagnostic procedures of two psychiatrists. The levels of passionate love of all of the volunteers were scaled using the Passionate Love Scale (PLS),^4^ and they had an interview with a certified psychologist. We included the couples who had fallen in love with each other not more than 100 days. All of the subjects agreed to participate in this experiment voluntarily and provided their informed consent to participate in the study and to appear on the nationally televised documentary film, "Love". The Institutional Review Boards (IRB) at The Catholic University of Korea approved all of the experimental procedures.

### Passionate love scale

The PLS is a 15- or 30- item Likert type scale, designed to measure the level of passionate love, which is defined as "a state of intense longing for union with another". It taps the cognitive, emotional, and behavioral indicants of passionate love. We used the short 15-item scale. Each item scores from 1 to 9, so the highest total score is 135. This evaluation was made before the fMRI scanning at the baseline and after 6 months.

### Experimental design

Photo strips of various facial expressions of the participant's lover, friends of the same sex, blurred human faces, and a gray background without face were made as visual stimuli for each subject. The friends of the same sex were the oldest and closest ones chosen by the participants. They all had the same age as their friend and their friendship existed for at least 5 years.

To make the photo strips, all of the subjects were asked to bring friends of the same sex and their lovers to the broadcasting studio, and all of the photos were taken at the same place, at the same time and under the same conditions. The task paradigm was as follows: 5 still photos depicting various facial expressions of a friend of the same gender running for 30 seconds, blurred human faces for 30 seconds, 5 still photos portraying different facial expressions of the lover displayed for another 30 seconds, and finally a gray background without face for the last half minute. This six-minute sequence was repeated to form a 12-minute movie clip. We performed the fMRI experiment and the PLS twice at the end of May and the beginning of December, 2005 (i.e. with a 6-month interval between the two).

### Image acquisition

Echo-planar images were collected on a 1.5 T MRI scanner (Magnetom Vision, Siemens, Erlangen, Germany) using a standard head coil and Siemens Magnetom gradient overdrive. For each subject, four time series (friend, blurred human face, lover and no object against gray background) of the whole-brain images were obtained with a gradient-echo and echo-planar scanning sequence (EPI; TR 3 s, TE 60 ms, flip angle 90°; FOV 240 mm^2^, acquisition matrix 64×64 , 30 axial slices, slice thickness 5 mm, gap 0 mm). The first five images were discarded to account for spin saturation effects. A T1-weighted 3D magnetization prepared rapid acquisition gradient-echo sequence (MP RAGE) scan (voxel size=1.0×1.0×1.0 mm^3^) lasting 7 minutes and 14 seconds was recorded in the same session as a functional measurement for the recording of the subject's individual brain anatomy.

### Data processing

Pre-processing and statistical analysis of the fMRI data was performed using Statistical Parametric Mapping software (SPM99; Wellcome Department of Cognitive Neurology; http://www.fil.ion.ucl.ac.uk/spm). For motion correction, all T2 weighted volumes were realigned to the first in the time series. All data for the participants was unwrapped. The images were normalized to standardized MNI space and then smoothed with an 8 mm full width at half maximum (FWHM) isotropic Gaussian kernel. A high pass filter was applied to remove low frequency fluctuations in the blood oxygen level dependent (BOLD) signal. For random effects analysis, we used related sample t-tests to examine separately the significance of the BOLD responses during the presentation of the targets (lover's photo or friend's photo) relative to the background stimuli within each group. The subsequent between group t-tests demonstrated the existence of significant differences between the groups. The data was initially analyzed using whole-brain analysis to explore the brain networks significantly activated compared to the standard tones within each group, and to explore between-group differences at the whole brain level. A statistical threshold of p<0.05, uncorrected for multiple comparisons and an extent threshold greater than or equal to 200 voxels per cluster, was used to determine significant activations in the whole-brain analysis.

## Results

### Temporal changes in passionate love scale

We estimated the degree of romantic love for the subjects using the PLS and professional interviews twice at an interval of 180 days (at the end of May and the beginning of December, 2005). During the interviews, the participants stated that their love would undoubtedly last for a long time. This passionate feeling was supported by the corresponding PLS scores. We found high PLS scores (M: 118.6±9.1, F: 120.2±7.0) at the early stage of intense love. This indicates that the couples were all deeply and passionately in love. However, after 180 days, we found that their PLS values were decreased (M: 110.8±4.0, F: 106.2±3.0), although this reduction was not statistically significant. All of the couples stated that their relation remained unchanged during the 180 days, but three out of ten participants told us secretly that they had sometimes considered separation during this time.

### Neural activity at an early stage of romantic love

**FIGURE 1:**
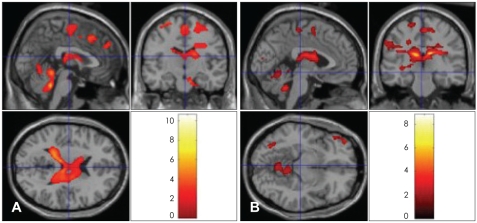
Neural activation sites for romantic-love related stimuli (A) at the early stage within 100 days from the first meeting (May 2005) and (B) in 6 months (December 2005).

From the BOLD signal obtained by subtraction of the neural response that occurred while the subjects were shown pictures of their lovers and viewing their friends, we found statistically significant activations at an early stage of love. The left superior frontal gyrus, left medial frontal gyrus {Brodmann area (BA) 6}, left cingulate gyrus (BA 32), left subgyrus, right pons and right precentral gyrus, right frontal gyrus, right parietal postcentral gyrus, and anterior lobe of right cerebellum were significantly activated (p<0.05) when the participants were viewing the pictures of their lovers, compared with when they were viewing the pictures of their friends. Among these activations, those which reached the most significant level were the left superior frontal gyrus, left cingulate gyrus (BA 32), and anterior lobe of the right cerebellum (p<0.001)(Table 1, Figure 1A). This indicates that these regions are involved in the neural process at the early stage of romantic love.

### Neural activities 180 days after the preliminary scan

The same paradigm was used at the second scan 180 days after the initial fMRI scanning. Significant activations were found in this experiment at the corpus callosum, left inferior frontal gyrus, left middle frontal gyrus (BA 11), left middle frontal gyrus, right medial frontal gyrus (BA 6), right temporal gyrus, left superior frontal gyrus, right parietal postcentral gyrus (BA 3), right middle occipital lobe, and certain areas of the cerebellum (p<0.05) when the participants were viewing the pictures of their lovers, compared with those when they were viewing the pictures of their friends. Particularly, the corpus callosum reached the most significant level of activation (p<0.001)(Table 2, Figure 1B). All of these regions are consistent with the neural correlates of romantic love at the earlier stage, except for the corpus callosum (Figure 1).

**FIGURE 2:**
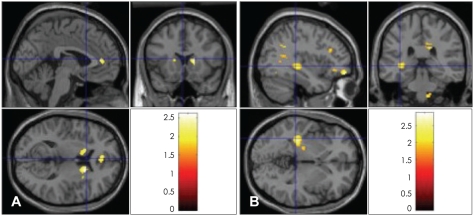
Temporal changes in (A) neural activations and (B) deactivations between initial fMRI measurement and the measurement in 6 months. fMRI: functional magnetic resonance imaging.

The neural activity patterns between the two measurements (May and December, 2005) were compared. We found that the left middle frontal gyrus, right inferior frontal gyrus, both cingulate gyrus areas (BA 31 and BA 33), the corpus callosum, both parietal supramarginal gyrus areas, both parietal precuneus areas and the left middle occipital gyrus (BA 18) showed significantly increased levels of activation when the participants were viewing the pictures of their lovers after 180 days (December 2005) compared with those measured initially (May 2005; p<0.05)(Table 3, Figure 2A). On the other hand, the head and tail of the right caudate were found to be less activated when the participants were viewing the pictures of their lovers after 180 days as compared to the initial measurement under the same conditions (p<0.05)(Table 4, Figure 2B). These results suggest that temporal alterations in romantic love are associated with the limbic regions and are connected to the frontal gyri.

## Discussion

The aim of this study was to investigate the neural systems underlying the later stages of romantic love as compared to its early stages. To the best of our knowledge, this is the first study to carry out a follow-up fMRI scan in order to investigate the temporal changes in the neural activity of enduring love. In this comparison with an interval of 180 days for the same romantic couples, we found that the activation of the caudate was significantly reduced, while that of the cortical regions including the cingulate gyrus was increased when the participants were viewing the pictures of their loved ones after 180 days, as compared with the initial measurements. Their PLS values were also significantly reduced after 6 months. This result suggests that the anterior cingulate gyrus and the caudate nucleus are likely the major regions involved in romantic love and that they exhibit a dynamic alteration over time as the degree of their romantic love evolves. This finding supports the hypothesis that romantic love is a dynamic process in the brain.

The early stage of romantic love in this study is marked by activations in the left superior frontal gyrus, left cingulate gyrus, left subgyrus, and sub-lobar regions, and the anterior lobe of the right cerebellum, while the participant is viewing his or her lover's face as compared to those of his or her friends. The left cingulate gyrus, which is located in the dorsal region of the anterior cingulate, is reportedly associated with various emotional states including happiness and pain.^16^ The anterior cingulate cortex is also known to be involved in conditioned emotional learning and internal state representations.^17^ Bartel and Zeki^5^,^6^ obtained similar results in their previous studies, in which the anterior cingulate gyrus was more activated when the participants were viewing their lover's picture than when they were viewing those of their friends. The cingulate gyrus has rich connections with subcortical areas, including the caudate, and with prefrontal cortex and maternal-infant interactions, as well as regulating autonomic and endocrine functions. Thus, the results of this study are likely consistent with those of previous studies which reported that the neural correlates of intense emotional states and social behavior are possibly related with romantic love, as compared with friendship with the opposite gender. We found in this study that the left superior frontal gyrus, part of the lateral prefrontal cortex, was activated when the participants were viewing their lover's picture. However, Bartel and Zeki^5^,^6^ found that the right superior frontal gyrus was deactivated in a similar experimental paradigm. We speculate that activation in the left superior frontal gyrus may be related to the deactivation of the right superior frontal gyrus, due to their functional connectivity and reciprocal anatomical interaction.

The most significant finding of this study is that the neural correlates of romantic love change over time. It is commonly known that the fire of passionate love cools down over time. Changes in neural activation associated with romantic love over time have previously been suggested.^2^,^3^ These previous studies showed that the activation of the right anterior and posterior cingulate, as well as that of the right mid-insular cortex, was positively correlated with the duration of the romantic relationship.

The significant decrease in fMRI activation in the head and tail of the right caudate nucleus after 180 days is consistent with previous studies which reported that the caudate nucleus is an important region in romantic love.^5^,^6^ This finding of the present study suggests that the attenuation of romantic love is associated with a decrease in neural activity of the head and tail of the caudate nucleus. The caudate nucleus is one of the most commonly reported activation regions related to various emotions, sexual response, and multi-modal information processing and inhibition. After 6 months, the caudate can exert modulatory effects on motor activities and facial gestural posture and aid in the maintenance of selective motoric attention via inhibition characterized by a decrease in cerebral blood flow. Lesions, destruction, or shrinkage of the head of the caudate nucleus in humans can result in sensory neglect, agitation, hyperactivities and distractibility.^18^-^21^ Left caudate lesions are more likely to result in bipolar mania,^18^ and right caudate hypermetabolism and blood flow are associated with obsessive-compulsive disorders (OCD).^22^ This is interesting when considering that the passion of love can resemble a manic-like psychosis in terms of its flamboyance and escape from reality. Also, OCD-like behavior was observed in romantic couples, for instance focusing attention on each other and/or thinking obsessively about their partners. The caudate nucleus has also been revealed to play major roles in reward and motivation in the mammalian brain.^23^,^24^ Therefore, our results suggest that as passionate love decreases over time from its initial high level at the early stage, the dopamine-rich subcortical reward regions gradually exhibit less activation, resulting in reduced motivation and reward.^2^,^3^

Our results show the cingulate gyrus to be more activated after 180 days than at the initial measurement, which is consistent with the previous finding of larger activation in the cingulate gyrus for couples having longer durations of love.^3^ The cingulate gyrus is a part of the limbic cortex, which interconnects the limbic areas with the cortical regions, and which deals with both emotion and cognition.^21^ The cingulate gyrus and limbic cortex have efferent connections with the subcortical areas, including the caudate and prefrontal cortex. Furthermore, many important aspects of the anterior cingulate cortex have come to be understood through the elucidation of the patterns of its efferent and afferent connections.^17^ Thus, our results suggest that romantic love is importantly associated with the limbic cortex, i.e., the dorsal (paralimbic) tier deeply buried in the cingulate sulcus. This is consistent with a previous finding.^5^ Even after 180 days, the anterior cingulate gyrus was still at the center of the activation for romantic love.

Previous fMRI studies on romantic love used participants heterogeneous in age and the duration of love. For instance, Aron and his colleagues^2^ included 17 participants exhibiting a broad range of durations of being in love from 1 to 17 months. The current study suggests that, because romantic love is a dynamical neural process which evolves over time, a homogenous group of subjects in terms of the duration of being in love should be used in the future in order to obtain more reliable results.

## Figures and Tables

**TABLE 1:**
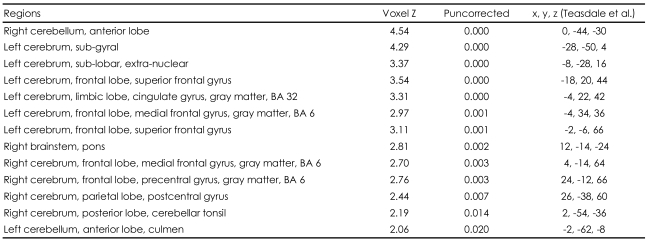
Neural activation sites for romantic-love related stimuli at the early stage within 100 days from the first meeting (May 2005)

**TABLE 2:**
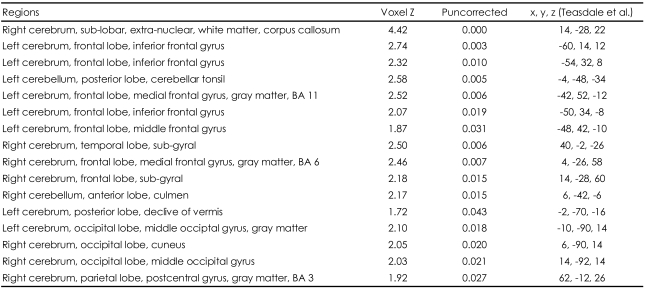
Neural activation sites for romantic-love related stimuli in 6 months (December 2005)

**TABLE 3:**
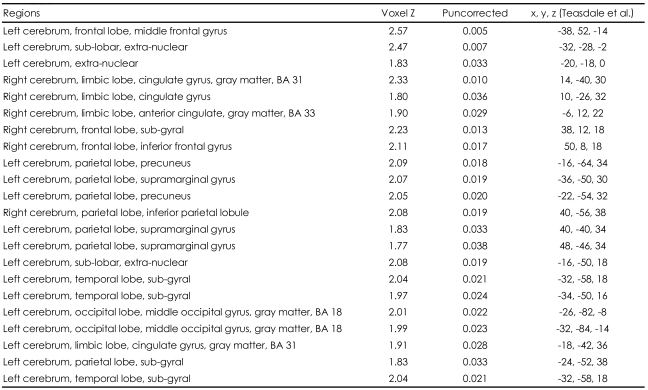
Temporal changes in neural activations between initial fMRI measurement and the measurement in 6 months

fMRI: functional magnetic resonance imaging

**TABLE 4:**
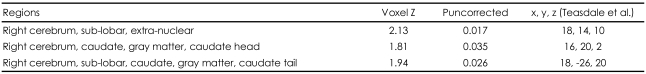
Temporal changes in neural deactivations between initial fMRI measurement and the measurement in 6 months

fMRI: functional magnetic resonance imaging
